# Genetic parameters for direct and maternal genetic components of calving ease in Korean Holstein Cattle using animal models

**DOI:** 10.5713/ab.24.0281

**Published:** 2024-10-07

**Authors:** Mahboob Alam, Jae-Gu Lee, Chang-Gwon Dang, Seung-Soo Lee, Sang-Min Lee, Ha-Seung Seong, Mina Park, Jaebeom Cha, Eun-Ho Kim, Hyungjun Song, Seokhyun Lee, Joonho Lee

**Affiliations:** 1 Animal Breeding and Genetics Division, National Institute of Animal Science, Rural Development Administration, Cheonan 31000, Korea; 2 Dairy Cattle Improvement Center of NH-Agree Business Group, NACF, Goyang 10292, Korea; 3 GENEAPPS, Seoul 06105, Korea

**Keywords:** Animal Model, Calving Ease, Direct Effect, Genetic Parameter, Korean Holstein, Maternal Effect

## Abstract

**Objective:**

We investigated genetic parameters of calving ease (CE) using several animal models in Korean Holstein and searched for suitable models for routine evaluation of CE.

**Methods:**

Two phenotypic datasets of CE (DS5 and DS10) on first-parity Korean Holstein calves were prepared. DS5 and DS10 included at least 5 and 10 CE records per herd-year level and comprised 117,921 and 80,389 observations, respectively. The CE phenotypes ranged from 1 to 4, from a normal to extreme difficulty calving scale. The CE was defined as a trait of the calf. The BLUPF90+ software was used for (co)variances estimation through four animal models with a maternal effect (M1 to M4), where all models included effects of a fixed calf-sex, a fixed dam calving age (covariate), and one or more fixed contemporary group (CG) terms. The CG effects were different across models—a herd-year-season (M1, HYS), a herd-year and year-season (M2, HY+YS), a herd-year and season (M3, HY+S), and a herd and year-season (M4, H+YS).

**Results:**

Direct heritability (h^2^) estimates of CE ranged from 0.005 to 0.234 across models and datasets. Maternal h^2^ values were low (0.001 to 0.090). Genetic correlations between direct and maternal effects were strongly negative to lowly positive (−0.814 to 0.078), further emphasizing its importance in CE evaluation models. These genetic parameter estimates also indicate slower future selection progress of CE in Korean Holsteins. The M1 fitted many levels with fewer observations per level deriving unreliable parameters, and the M4 did not account for confounded herd and animal structures. The M2 and M3 were deemed more realistic for implementation, and they were better able to account for data structure issues (incompleteness and confounding) than other models.

**Conclusion:**

As the pioneering study to employ animal models in Korean Holstein CE evaluation, our findings hold significant potential for this breed’s future and routine evaluation development.

## INTRODUCTION

The prevalence of a difficult birth (dystocia) or calving ease (CE) is a growing concern in Korean Holstein cows that can exert adverse effects on farm economics. Alongside some short-term economic consequences due to loss of calf, death of dam, veterinary and extra labor fees, other long-term consequences related to undesired animal health issues, cow fertility problems, reduced production, and increased culling rate are also highly likely in farms [1,2]. Reports suggest that cows receiving calving assistance can have subsequent fertility and productivity problems. According to Dematawewa and Berger [1], the impact of dystocia can be substantial on production (41% of costs), fertility (34%), and morbidity and mortality of cow and calf (25%). Other adverse effects (culling, calf loss, digestive disorders and so on) have also been reported in many studies [3,4]. This indicates that genetic improvement of CE in dairy cattle is a necessity due to a range of potential risk concerns.

Generally, direct and maternal genetic components are considered responsible for CE phenotypes from the genetic standpoint [5]. The direct component expresses genetic ability of a calf to be born easily. In contrast, the maternal component describes the ability of a female calf as a dam to give birth easily. Meijering [6] earlier discussed the biological aspects of the relationship between these two genetic components. According to their suggestions, female calves of small dimension are not only likely to be born easily but also prone to express more calving difficulties as dams due to their reduced pelvic dimensions. In contrast, a male calf is also more likely to experience calving difficulty due to its higher birth weight than a female calf. Therefore, it is also essential to understand these genetic components and their relationship in a dairy population.

However, the ordered-categorical nature of the phenotype imposes a challenge for genetic evaluations, especially regarding the choice of analysis model. Many earlier studies have applied linear models [7,8] and threshold models [9]. A logistic transformation of the trait by Snell procedure has also been used in linear model-based studies [10]. Threshold models are often considered more suitable for categorical traits [11]. However, arguments supporting linear model fit have some practical scenarios, such as in a population with relatively smaller contemporary groups (CGs) or sire group sizes where such a linear model could perform better [12]. We will perform a linear model fit with CE in this study as the Korean Holstein population resembles somewhat closer to the latter scenario. Also, an animal model could also be a model of choice for routine national evaluations due to its simplistic nature and its ability to include more animals in a given animal population.

Despite an assumed complex genetic nature of CE, many previous studies [2,9] have reported evidence of adequate genetic variances to allow practical and effective selection. However, developing a statistical model of CE for national-level routine evaluation may take more work and will be different from other dairy and beef cattle populations. It is because the definition of CE phenotype (i.e., number of levels) is generally subjective and have large variations across countries [3]. Therefore, selecting a national evaluation model might demand a careful balance between several factors, such as the model’s prediction ability, ease of use, and computational feasibility [13].

For Korean Holstein, only a few reports are available regarding their genetic potential [14,15]. The objective of this study was to investigate genetic parameters for direct and maternal effects of CE using multiple animal models and study models’ feasibility in Korean national genetic evaluation programs.

## MATERIALS AND METHODS

### Animals, phenotype, and pedigree data

This study considered CE scores from the first parity of calf birth events between 2002 and 2024 for Korean Holstein cattle. Phenotype data were provided by the Dairy Cattle Improvement Center (DCIC) of Korea. Under the Korean National Evaluation System, CE consists of four ordered categories (1 to 4) based on the observed level of difficulty at calf birth. The difficulty level was measured by the degree of assistance provided during calving. A CE of 1 indicated a normal calving event without help at birth (non-assisted). In contrast, a score of 4 indicated veterinary assistance (extreme difficulty at birth requiring surgical help). Intermediate scores (i.e., 2 and 3) indicated the total number of personnel assisted during calving: slightly assisted calving (assisted by one person) and difficult calving (assisted by two or more persons), respectively. We defined CE as a trait of calf (progeny), with an assumption that calf’s physical dimensions and birth weight play a major role in most calving problems. This trait definition would enable the evaluation of the direct contribution of sires in subsequent progeny generations.

We applied several data filters to obtain the final datasets. Animals (calves) were required to have valid parental information. Information of multiple births (twins and triplets) was discarded. Datasets only included observations associated with respective gestation lengths of 260 to 305 days of dams. More restrictions on the parity 1 dataset included dams’ calving ages residing within 20 to 42 months. We also removed farm records containing only normal calving to avoid any possible data recording bias by farmers. Using pedigree and birth information of animals and calving information of dams, we traced calf IDs for respective calving records. However, many male and some female calves’ identification numbers were missing, even though their valid sire and dam information and other essential information were available. Therefore, we assigned dummy IDs to those calves to avoid their exclusion from analysis. Afterwards, we put a restriction on the number of observations per HY level and prepared two different datasets such as DS5 (≥5 obs. per HY) and DS10 (≥10 obs. per HY) to study the effects of CG sizes on genetic analyses of animals. A total of 117,921 and 80,389 calf records were remained in DS5 and DS10 datasets, respectively, for further analyses. These final datasets (see more in Table 1) consisted of information on the sex of the calf (SEX), dam’s calving herd, dam’s calving year, dam’s calving season, and dam’s calving age (DCA). Four seasons of calving (i.e., spring, March to May; summer, June to August; autumn, September to November; and winter, December to February) were considered. We prepared the related animal pedigree files for each dataset from a pedigree database provided by another association, the Korea Animal Improvement Association (KAIA), which is responsible for managing the dairy cattle pedigree databases in the country. The pedigree for DS5 and DS10 datasets comprised 317,126 and 233,569 animals, in which animals were traced back up to 23 generations.

### Data analysis

We estimated (co)variance components and genetic parameters for CE trait through animal models using the first parity dataset. We presented four univariate animal models for genetic parameter estimation. All four models included a random dam (maternal) effect, which was also assumed to be correlated with the random animal (direct) component. However, maternal permanent environment effect was ignored because of the single parity data structure. The SEX was a fixed effect across models, whereas the DCA (in days) was a fixed covariate effect in models to control for the effect of different ages of dams on CE. The CG effects *e.g*., dam’s calving herd, year, and season were considered as fixed effects but fitted differently across four models. To illustrate, Model 1 (M1) considered a combined fixed effect of the former three effects as herd-year-season (HYS). In model 2 (M2), the combined herd-year (HY) and year-season (YS) effects were fitted. Model 3 (M3) included a HY effect and a season (S) term as an independent effect. In model 4 (M4), herd (H), and YS effects were fitted. The reason for CG variations was to examine the influence of CG structures on variance component estimation. The BLUPF90+ software package [16] was used to estimate variance components through the average-information REML algorithm, genetic parameters, and their standard errors (SEs). The linear mixed model of the animal model with maternal effect in matrix notation was as follows:


$$y=Xb+Z_{d}d+Z_{m}m+e$$

where *y* was the vector related to CE; *b* was the vector of fixed effects, i.e., SEX, DAC, HYS or others (H, S, YS, HY etc.); *d* was the vector of random animal (direct) effect; *m* was the vector random maternal effect; and *e* was the vector of random residual effect. **X**, **Z_d_**, and **Z_m_** were design matrices relating effects to the phenotype. A covariance structure for random effects was assumed as follows:


$$\text{var}\;\left[\begin{array}{c} d\\ m\\ e \end{array}\right]=\left[\begin{array}{ccc} A\sigma_{d}^{2} & A\sigma_{dm} & 0\\ A\sigma_{md} & A\sigma_{m}^{2} & 0\\ 0 & 0 & I\sigma_{e}^{2} \end{array}\right]$$

where $\sigma_{d}^{2}$ was the direct genetic variance, $\sigma_{m}^{2}$ was the maternal genetic variance, $\sigma_{e}^{2}$ was the residual variance, and σ*_dm_* was the covariance between direct and maternal genetic effects. Therefore, the genetic covariance matrix (**G_0_**) between *d* and *m* was:


$$G_{0}=\left[\begin{array}{cc} \sigma_{d}^{2} & \sigma_{dm}\\ \sigma_{md} & \sigma_{m}^{2} \end{array}\right]$$

Total phenotypic variance ( $\sigma_{p}^{2}$) and different heritability estimates (direct, ${\text{h}}_{d}^{2}$; maternal, ${\text{h}}_{m}^{2}$; and total, T^2^), direct-maternal genetic correlation (r*_dm_*) [17] were calculated using the above (co)variance components as follows:


$$\begin{array}{l} \sigma_{p}^{2}=\sigma_{d}^{2}+\sigma_{dm}+\sigma_{m}^{2}+\sigma_{e}^{2}\\ {\text{h}}_{d}^{2}=\frac{\sigma_{d}^{2}}{\sigma_{\text{p}}^{2}},\\ {\text{T}}^{2}=\frac{\sigma_{d}^{2}+\sigma_{m}^{2}+2\sigma_{dm}}{\sigma_{p}^{2}}, \end{array}$$

and ${\text{r}}_{dm}=\frac{\sigma_{dm}}{\sqrt{\sigma_{d}^{2}\times \sigma_{m}^{2}}},$

Here, ${\text{h}}_{\text{d}}^{2},\;{\text{h}}_{\text{m}}^{2}$, and T^2^ expressed the ratio of direct, maternal and total heritable variances available for response to selection over phenotypic variance, respectively.

Approximated SEs of genetic parameters were obtained from (co)variance components using the BLUPF90+ software package, in which a Monte Carlo method was implemented for computation of SE following the study of Houle and Meyer [18]. We plotted the distribution and genetic trends of predicted direct and maternal estimated breeding values (EBVs) using the ‘ggplot2’ package in R [19]. We also calculated the mean squared error (MSE) statistic of these models using predicted phenotype estimates for each model and dataset. In the following sections, we will mainly emphasize on direct and maternal heritability estimation and the genetic correlation between direct and maternal components. Total heritability estimates are provided for completeness purpose only.

## RESULTS AND DISCUSSION

### Heritability, variance component and genetic correlation estimates

In this study, we investigated two datasets of CE (DS5 and DS10) on calves, which were born from the first parity of Holstein cows. Table 2 presents (co)variance and genetic parameters estimates for direct and maternal genetic components of CE from four different animal models with a maternal effect. Across models, the estimated ${\text{h}}_{\text{d}}^{2}$ parameter ranged from 0.005±0.002 to 0.168±0.012 within DS5 animals and from 0.014±0.005 to 0.234±0.018 within DS10 animals. Estimates of maternal heritability ( ${\text{h}}_{\text{m}}^{2}$) were lower than ${\text{h}}_{\text{d}}^{2}$ from all models using both datasets. We found that ${\text{h}}_{\text{m}}^{2}$ ranged from 0.001±0.002 to 0.090±0.007 within studied animals. In both datasets, M4 model derived much higher ${\text{h}}_{\text{d}}^{2}$ estimates than first three models, which is also similar to ${\text{h}}_{\text{m}}^{2}$ estimates. This study also showed somewhat similar or slightly lower total heritability (T^2^) estimates than other heritability estimates from DS5 and DS10 analyses, where obtained T^2^ ranges were from 0.007 to 0.018 across datasets. Like other h^2^ estimates, first three models (M1 to M3) also derived slightly lower T^2^ estimates than M4 model. Although most random (co)variance estimates were generally lower in this study, those from M1, M2, or M3 model appeared shrunk by some magnitude compared to the M4 model. Genetic correlation estimates between direct and maternal effects (r_dm_) were mainly negative except for the slightly positive value (r_dm_, 0.078±1.72) from M1 model using DS5 dataset. For DS10, r_dm_ estimates were moderate to strongly negative (−0.814±0.169 to −0.491±0.047). Overall, most results indicated negative correlation between direct and maternal genetic components of CE.

The knowledge of various genetic effects, such as direct and maternal effects and their relationship, is the key to CE improvement for any dairy cattle. In this study, we demonstrated that heritability (h^2^) estimates for CE in Korean Holsteins ranged from very small to low. In similar Korean Holsteins, a previous study using a sire-maternal grandsire (S-MGS) model for parity-1 progeny [15] revealed direct h^2^ (0.11) and maternal h^2^ (0.05) estimates, somewhat close to those of ours with M4 model estimates. Our results also partially agree with the study by Eaglen and Bijma [17] in Dutch Holstein-Friesian, showing their h^2^ estimates (direct, ~0.08; maternal, ~0.04) in our estimation range. Mujibi and Crews [10] have reported h^2^ estimates (direct, 0.14; maternal, 0.06) for Charolais cattle, also in agreement with ours. For those of very low h^2^ estimates in this study, multiple studies from Iranian Holstein cattle [20,21] provide support with their very small direct (0.02 to 0.041) and maternal h^2^ (0.002 to 0.012) estimates. Similar low h^2^ for direct (0.03) and maternal CE (0.02) were also available from Eaglen et al [13]. However, Roughsedge et al [22] using a linear mixed model indicated a wide range of direct h^2^ among beef breeds (0.13 to 0.35), aside from their agreeable maternal h^2^ range (0.07 to 0.11). Differences among reports are mainly due to differences in fitted factors, model types, trait definitions, breeds, etc. According to some studies [8], linear models tend to yield lower estimates than threshold models. Salimi et al [20] have argued that their large phenotypic variances (or residual variances) compared to genetic variances are possibly arising from their recording methods and herd management practices, inevitably underestimating their population’s direct and maternal components. Eaglen and Bijma [17] have also argued that whether the model is an animal or S-MGS model, some h^2^ parameters (e.g., maternal h^2^) are prone to inaccurate estimations despite having a sufficiently large dataset.

**Figure 1:**
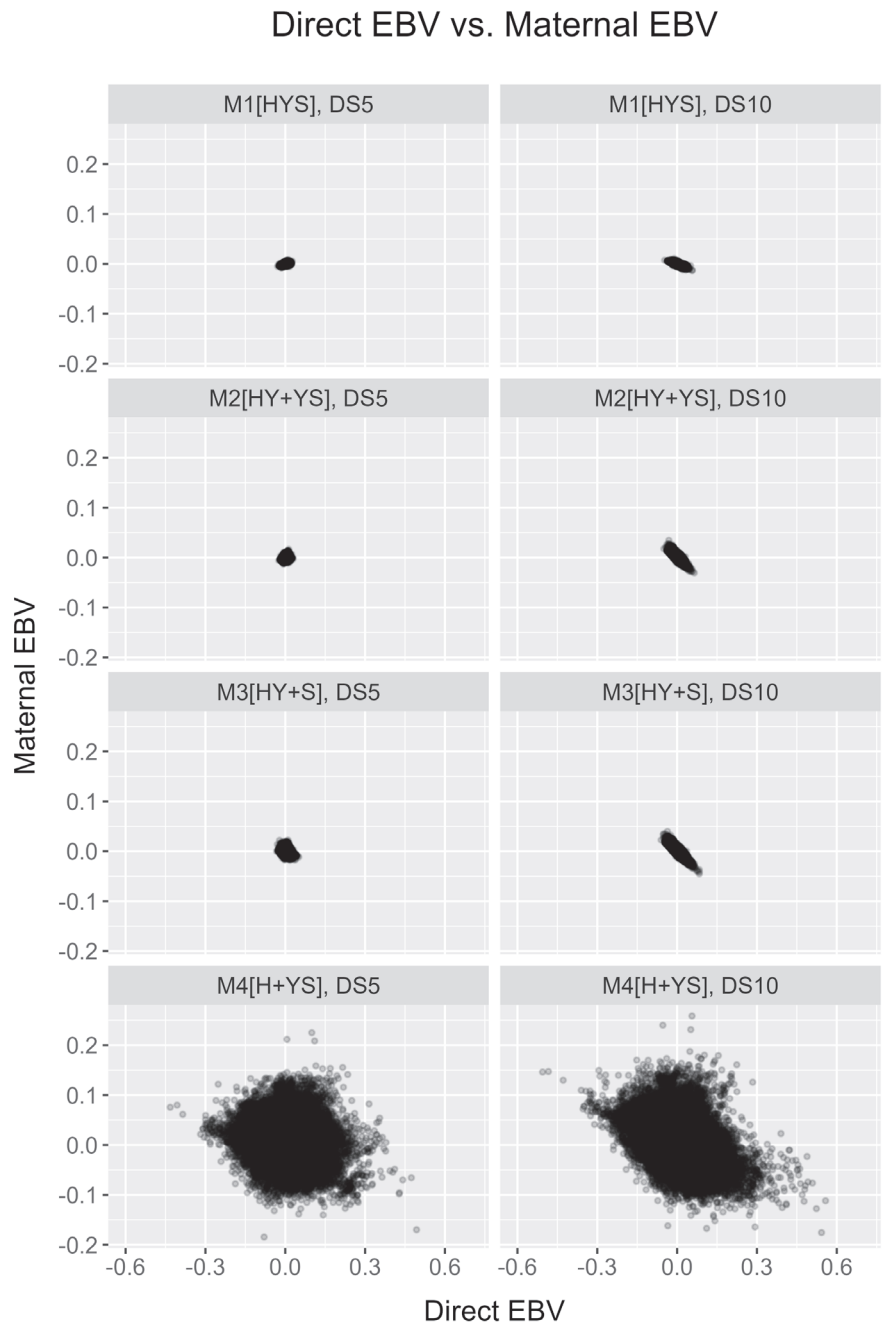
Distributions of direct EBV and maternal EBV of calving ease in Korean Holstein using four animal models (M1 [HYS], a model having calving herd-year-season effect; M2 [HY+YS], a model having calving herd-year and year-season effects; M3 [HY+S], a model having calving herd-year and season effects; M4 [H+YS], a model having calving herd and year-season effects) and two datasets (DS5, a dataset with ≥5 records per HY level; DS10, a dataset with ≥10 records per HY level). EBV, estimated breeding value.

In the present study, the genetic correlation estimates between direct and maternal effects varied across models and datasets. Most models indicated a negative association between the two genetic components. The distribution of these effects among animals also supports this negative association (Figure 1), despite some dataset related variations. Overall, our r_dm_ estimates ranged from lowly negative to strongly negative. Previous reports provide good overall support for our observed negative correlation estimates. Alam et al [15] have used an S-MGS model in Korean Holstein and reported a genetic correlation (−0.68), which supported the present study. Salimi et al [20] and Ghiasi et al [21] have reported somewhat comparable r_dm_ (−0.41 to −0.43) estimates in Iranian Holstein. The r_dm_ estimates from the study of Eaglen and Bijma [17] also showed agreeable estimates using an animal model (−0.04 to −0.44). Some agreements in Holstein cattle by a near absence of correlation (i.e., weakly negative to weakly positive) are also available [23,24]. The r_dm_ estimate varied widely in the literature, and multiple factors could be involved in such variabilities. First, it could be due to differences in breed or population in studies such as beef cattle, in which correlation estimates are often highly negative [25]. Second, a possible estimation bias is also likely to genetic covariance between direct and maternal effects [26].

To further discuss the estimated genetic parameters, we argue that future genetic improvements for CE in Korean Holsteins using an animal model (with maternal effects) could be slower due to lower h^2^ values, similar to any other reproductive trait. Given the increased calving difficulties in Korean Holstein cattle, the higher ${\text{h}}_{\text{d}}^{2}$ than ${\text{h}}_{\text{m}}^{2}$ indicates a greater contribution of sires for CE (through calf weight and calf dimension). In this study, we defined CE as a trait of the calf, which further indicates that present evaluation models will essentially allow relatively slender or less broad calves (any sex) to be born easily but finally cause more difficulties for female calves when they give birth as dams (due to reduction in pelvic dimensions) [13]. In this regard, a negative r_dm_ indicates the importance of the maternal component in CE evaluation. Our r_dm_ values suggest that selection improvement in the first parity animals could exert challenges due to such negative associations. Therefore, considering direct effects only for CE improvement (with an ignored negative correlation between direct and maternal genetic components) can eventually reduce the selection progress [27].

This study also revealed some additional challenges regarding data structure in Korean Holsteins. With animal models, genetic parameters were relatively sensitive to data structure changes in studied animals, indicating data connectedness problems with higher-order HY or HYS effects in the model. Our further investigation into datasets suggests that herds and dams are likely confounded with each other to some extent as dams and their first calving daughters hardly changed their herds. Therefore, accurately estimating genetic parameters for CE in Korean Holstein might be challenging. Such evaluations could inflate genetic parameters due to inaccurate estimation of herd and genetic effects. Hence, careful consideration is required for CE parameter estimations. An alternative model to counter such possible herd confounding errors could be S-MGS models, where one sire’s progenies are more likely spread over many herds, which can be a topic for future studies.

### Genetic trends of direct and maternal components

**Figure 2:**
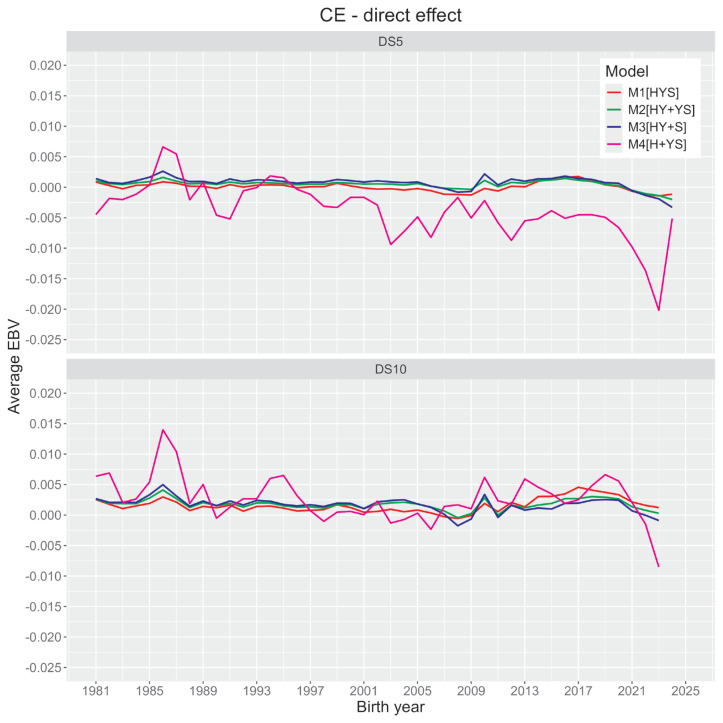
Trends of the average direct effect of calving ease (CE) trait in Korean Holstein obtained from four animal model analyses (M1 [HYS], a model having calving herd-year-season effect; M2 [HY+YS], a model having calving herd-year and year-season effects; M3 [HY+S], a model having calving herd-year and season effects; M4 [H+YS], a model having calving herd and year-season effects) and two datasets (DS5, a dataset with ≥5 records per HY level; DS10, a dataset with ≥10 records per HY level).

**Figure 3:**
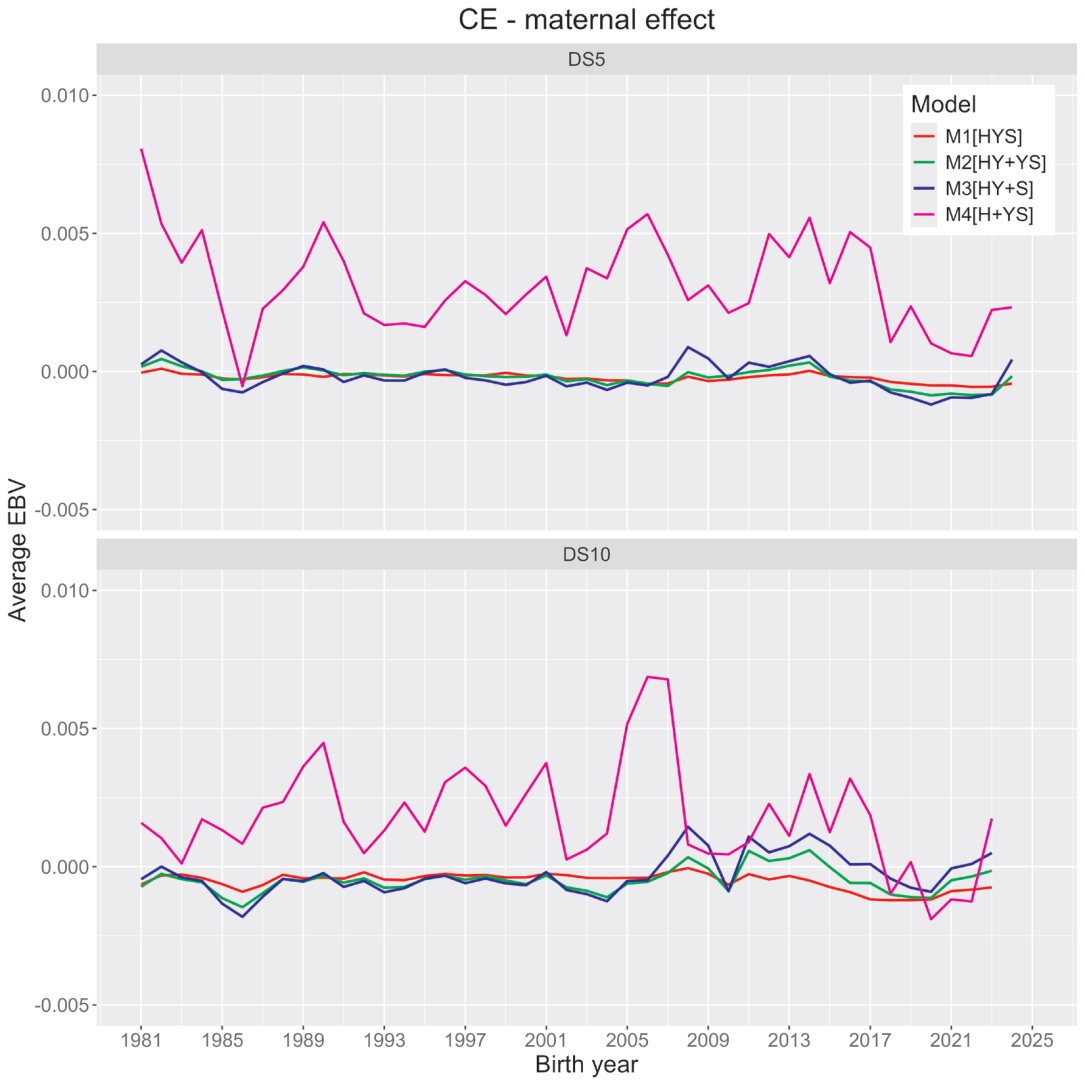
Trends of the average maternal effect of calving ease (CE) trait in Korean Holstein obtained from four animal model analyses (M1 [HYS], a model having calving herd-year-season effect; M2 [HY+YS], a model having calving herd-year and year-season effects; M3 [HY+S], a model having calving herd-year and season effects; M4 [H+YS], a model having calving herd and year-season effects) and two datasets (DS5, a dataset with ≥5 records per HY level; DS10, a dataset with ≥10 records per HY level).

Figure 2 and 3 illustrate changes in average direct and maternal genetic merit (EBV) of CE within Holstein cattle based on their birth year. All four models showed no significant trends for direct and maternal EBV estimates. However, the first three models (M1 to M3; herd as a combined effect) and the last model (M4; herd as an independent effect) had noticeable differences in EBV estimates (also see Figure 1). Average animal genetic effects trends from the former three models are less sensitive to yearly differences. In contrast, the last model showed noticeable yearly variations irrespective of datasets.

Most animal models in this study demonstrated negligible genetic improvement of CE. Contrary to our reports, an earlier report in Korean Holsteins using the S-MGS model [15] presented some improvements in direct and maternal EBVs. Such prediction disagreements could appear due to differences in our model from the previous report. For the M4 model, animals’ direct EBVs are deemed negative for an extended period (Figure 2), which is desirable. Using the direct-maternal relationship in Figure 1, we could suggest that their maternal EBV estimates were mostly positive or higher (Figure 3), which is also desirable. Although such desirable scenarios from Figure 2 and 3 could appear to be some improvements in CE over time, these trends using M4 need careful consideration. The M4 model could also have a potential confounding concern between some non-genetic effects and direct genetic effects. The confounded herds and animals in the datasets and the M4 model’s inability to correct for such data structures could potentially over- or under-estimate animal predictions.

### Candidate models for Korean national evaluation of calving ease

Table 2 also presents Akaike information criterion (AIC) estimates and MSE of predictions for each model fit obtained from BLUPF90+ analyses. We showed four linear mixed models following an order of more detailed to less detailed models and then compared their goodness-of-fit statistics. To illustrate, M1 was the most detailed model, which included the HYS effect (a 3-way interaction) and required higher computation requirements due to many levels fit, whereas M4 required the least computation requirements due to a relatively small number of levels fit. The M2 model was confirmed to have the best fit using DS10 and the second best fit model using the DS5 dataset. However, the M4 was a poorly fit model across datasets, despite the lowest model MSE estimates. Although M1 (with HYS) was the best-fit model using the larger dataset according to the AIC estimate, this model needs a careful interpretation due to the overall shrinkage of all variance components, especially with the maternal genetic component. Some possible reasons could be the lack of sufficient data per HYS level using M1 and the presence of disconnected data subsets within the primary dataset for specific factor combinations [28,29]. A possible fitting of data noises within disconnected subsets might not be unlikely due to the higher number of HYS levels with fewer records. The M4 model, which exhibits relatively higher (co)variances than other models, could potentially suffer from an ineffective separation of genetic effects from confounded herd effects. The genetic parameters estimated by M1 or M4 also require critical assessment. In this regard, M2 or M3 animal models or use of S-MGS model could be alternatives to account some of the challenges discussed above.

In contrast, for a routine national evaluation, designing a statistical model for maternally influenced traits should represent a careful balance of the model’s prediction ability and computational feasibility [12]. Note that the above factors might depend on several other factors, including the trait in question, dataset size, data recording biases, computing facility, and time availability for the task. From a simplicity standpoint, an animal model is preferable to other models (e.g., sire models) for routine evaluation of animals because more complicated models often have parameter convergence problems. Besides, an animal model is more appealing and practical, considering its ability to incorporate a large number of samples directly during animal evaluations. Therefore, we suggest considering models such as M2 or M3 in routine evaluation of Korean Holsteins, which could account for some existing data-related issues yet be practical.

## CONCLUSION

In this study, we evaluated the genetic potential of direct and maternal genetic components of CE in the first parity progenies of Korean Holstein. We also investigated candidate models for the Korean national evaluation of CE. Variance components and genetic parameters showed notable influences due to choices related to herd, year, and season group effects. The h^2^ estimates for direct and maternal effects ranged between very low to low values across datasets. The direct effect was more heritable than the maternal effect. The genetic correlation between direct and maternal genetic effects varied largely across datasets and models. Our substantial negative correlation estimates between the direct and maternal genetic effects suggest that female calves are likely to be born more easily, with a risk of simultaneously encountering more difficulty at giving birth as dams. Moderate to high correlation also signifies the importance of maternal effect for inclusion in the genetic evaluation models. Given the complexity and a large number of level fits by M1 or assumed difficulty separating herd effects from animal genetic terms by M4, we suggest a national evaluation model based on M2 or M3 that could account for the above limitations in Korean Holstein datasets. Our study using an animal model is the first report of the Korean Holstein population. Therefore, these estimates can significantly assist in making decisions for a routine evaluation of the CE trait in Korean Holsteins.

## Figures and Tables

**Table 1:** Structure of calving ease datasets on calves in the first parity of Korean Holstein^1)^

Factor/Term	Level	DS5	DS10
Total observation	-	117,921	80,389
Calf sex	Male	58,170	39,474
	Female	59,751	40,915
No. of calving herd (H) of dam	-	1,394	988
Calving year (Y) of dam	-	2002–2024	2007–2023
Calving season (S) of dam	Spring	30,000	20,353
	Summer	28,611	19,398
	Autumn	29,351	20,337
	Winter	29,959	20,301
Total sires	-	1,756	1,627
Total dams	-	117,921	80,389
No. of HYS level	-	37,226	20,047
No. of HY level	-	10,795	5,308
No. of YS level	-	77	66
Calving ease score	1	96,611	65,955
	2	20,856	14,131
	3	418	274
	4	36	29

1) DS5, a dataset with ≥5 records per herd-year level; DS10, a dataset with ≥10 records per herd-year level.

**Table 2:** Estimates of variance components, genetic parameters from animal models (with a maternal effect) using two calving ease datasets^1)^

Item	Model^2),3)^	$\sigma_{\text{d}}^{2}$	σ_dm_	$\sigma_{\text{m}}^{2}$	$\sigma_{\text{e}}^{2}$	$\sigma_{\text{p}}^{2}$	${\text{h}}_{\text{d}}^{2}\pm \text{SE}$	${\text{h}}_{\text{m}}^{2}\pm \text{SE}$	T^2^±SE	r_dm_±SE	AIC	MSE_P_
DS5												
	M1	0.0003	0.0000	0.0001	0.0618	0.0623	0.006±0.003	0.001±0.002	0.007±0.003	0.078±1.720	−542,993	1.521
	M2	0.0003	0.0000	0.0002	0.0677	0.0682	0.005±0.002	0.003±0.002	0.007±0.003	−0.116±1.526	−541,767	1.502
	M3	0.0006	−0.0002	0.0003	0.0679	0.0686	0.008±0.003	0.005±0.002	0.008±0.003	−0.414±0.402	−541,430	1.501
	M4	0.0165	−0.0029	0.0061	0.0786	0.0983	0.168±0.012	0.062±0.007	0.171±0.010	−0.288±0.055	−522,867	1.474
DS10												
	M1	0.0008	−0.0002	0.0001	0.0577	0.0585	0.014±0.005	0.002±0.003	0.010±0.004	−0.591±0.793	−405,446	1.516
	M2	0.0011	−0.0005	0.0005	0.0626	0.0636	0.017±0.005	0.007±0.003	0.007±0.003	−0.779±0.292	−408,817	1.501
	M3	0.0015	−0.0008	0.0006	0.0627	0.0641	0.024±0.005	0.010±0.004	0.009±0.003	−0.814±0.169	−408,595	1.500
	M4	0.0214	−0.0065	0.0082	0.0685	0.0916	0.234±0.018	0.090±0.010	0.181±0.013	−0.491±0.047	−394,895	1.479

$\sigma_{\text{d}}^{2}$, direct genetic variance; σ_dm_, covariance between direct and maternal genetic variance; $\sigma_{\text{m}}^{2}$, maternal genetic variance; $\sigma_{\text{e}}^{2}$, residual variance; $\sigma_{\text{p}}^{2}$, phenotypic variance; ${\text{h}}_{\text{d}}^{2}$, direct heritability; SE, standard error; ${\text{h}}_{\text{m}}^{2}$, maternal heritability; T^2^, total heritability; r_dm_, genetic correlation between direct and maternal effects; AIC, Akaike information criterion; MSE_P_, mean squared error for predicted EBVs of animals.

1) DS5, a dataset with ≥5 records per herd-year level; DS10, a dataset with ≥10 records per herd-year level

2) M1, model with the herd-year-season (HYS); M2, model with the herd-year and year-season (HY+YS); M3, model with the herd-year and season (HY+S); M4, model with the herd and year-season (H+YS); See Materials and Methods section for more model details.

3) Estimate of 0.0000 indicate value less than 0.00001.
